# A new anterior pharyngeal region specific fluorescent co-transformation marker

**DOI:** 10.17912/micropub.biology.000084

**Published:** 2019-01-16

**Authors:** Abhishek Bhattacharya, Oliver Hobert

**Affiliations:** 1 Department of Biological Sciences, Columbia University, New York, NY, USA; 2 Howard Hughes Medical Institute

**Figure 1.**
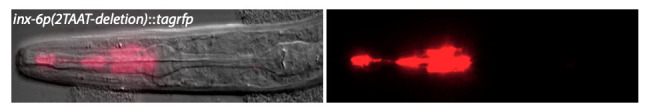
*inx-6p*(2TAAT-deletion)::tagRFP reporter (*otEx5854*) is only expressed in the procorpus region of the pharynx.

## Description

Among the fluorescence-based co-transformation markers, two of the most commonly used markers are pharyngeal expression of green fluorescent protein (GFP) and red fluorescent protein (RFP) under the *myo-2* *cis*-regulatory element (Frokjaer-Jensen et al., 2008; Tabara et al., 1996). However, bright expression of GFP or RFP in the posterior pharynx, i.e. in the isthmus and in the posterior pharyngeal bulb, restricts expression analysis of reporter genes that express fluorescent proteins with overlapping spectra in the head neurons. This shortcoming particularly limits the choice of fluorescent proteins that can be used when simultaneous imaging of multiple distinct reporter genes is required. We report here generation of a new fluorescence-based co-transformation marker that allows easy identification of transgenic animals based on bright TagRFP expression only in the anterior pharyngeal region (procorpus region) muscles at all developmental and adult stages (Figure 1) and does not interfere with the analysis of reporter gene expression in the head neurons.

This reporter expressed TagRFP under the regulation of 1654 bp *cis*-regulatory region upstream of the *inx-6* locus (Primers used to amplify the *cis*-regulatory region: upstream: 5’ cgataagattttgacgaatccg 3’ and downstream: 5’ tgtgaacaagctaaggagag 3’). We deleted two conserved putative homeodomain binding sites (TAAT) present within this region. Removal of the first binding site, at 422 bp from the start of the *cis*-regulatory region (TGTAATAC>TGAC) prevented reporter gene expression in the marginal cells. Deletion of the second binding site, at 521 bp (GATAATTA>GATA) prevented reporter gene expression in AIB interneurons during dauer and L1-diapause stages (Bhattacharya, 2018). We typically used 40ng/ul of circular reporter plasmid (pAB1) in microinjections. For complex arrays (Kelly et al., 1997), we used 6-8ng/ul of linearized pAB1.

## Reagents

OH12747 *otEx5854*[*inx-6p*(2TAAT-deletion)*::tagRFP* (8ng/ul); pRF4 (rol-6) (5ng/ul), OP50 gDNA (100ng/ul)]. Will be available at CGC.

pAB1: *inx-6p*(2TAAT-deletion)::*tagRFP*::*unc-54* 3’ UTR; contains 1654 bp region upstream of *inx-6* coding region, where two TAAT sites were deleted. Will be available at Addgene.
